# Raman Spectroscopy and Chemometric Modeling to Predict Physical-Chemical Honey Properties from Campeche, Mexico

**DOI:** 10.3390/molecules24224091

**Published:** 2019-11-13

**Authors:** F. Anguebes-Franseschi, M. Abatal, Lucio Pat, A. Flores, A. V. Córdova Quiroz, M. A. Ramírez-Elias, L. San Pedro, O. May Tzuc, A. Bassam

**Affiliations:** 1 Faculty of Chemistry, Autonomous University of Carmen, Street 56 No. 4 Esq. Av. Concordia, Col. Benito Juárez, Z. C. 24180 Ciudad del Carmen, Campeche, Mexico; fanguebes@pampano.unacar.mx (F.A.-F.); acordova@delfin.unacar.mx (A.V.C.Q.); mramirez@pampano.unacar.mx (M.A.R.-E.); 2 Faculty of Engineering, Autonomous University of Carmen, Campus III, Avenida Central s/n, Esq. Con Fracc. Mundo Maya, C. P. 24115 Ciudad del Carmen, Campeche, Mexico; mabatal@pampano.unacar.mx (M.A.);aflores@pampano.unacar.mx (A.F.);; 3 South Frontier College, Av. Rancho Polígono 2-A, Ciudad Industrial, 24500 Lerma, Campeche, Mexico; lpat@ecosur.mx; 4 Faculty of Engineering, Autonomous University of Yucatan, Av. Industrias no Contaminantes Periférico Norte, Cordemex, Z.C. 97310 Mérida, Yucatan, Mexico; liliana.cedillo@correo.uady.mx (L.S.P.); maytzuc@gmail.com (O.M.T.)

**Keywords:** quality control, Raman spectroscopy, honey, PLS regression models, physicochemical parameters

## Abstract

In this work, 10 chemometric models based on Raman spectroscopy were constructed to predict the physicochemical properties of honey produced in the state of Campeche, Mexico. The properties of honey studied were pH, moisture, total soluble solids (TSS), free acidity, lactonic acidity, total acidity, electrical conductivity, Redox potential, hydroxymethylfurfural (HMF), and ash content. These proprieties were obtained according to the methods described by the Association of Official Analytical Chemists, Codex Alimentarius, and the International Honey Commission. For the construction of the chemometric models, 189 honey samples were collected and analyzed in triplicate using Raman spectroscopy to generate the matrix data [X], which were correlated with each of the physicochemical properties [Y]. The predictive capacity of each model was determined by cross validation and external validation, using the statistical parameters: standard error of calibration (SEC), standard error of prediction (SEP), coefficient of determination of cross-validation (R^2^_cal_), coefficient of determination for external validation (R^2^_val_), and Student’s *t*-test. The statistical results indicated that the chemometric models satisfactorily predict the humidity, TSS, free acidity, lactonic acidity, total acidity, and Redox potential. However, the models for electric conductivity and pH presented an acceptable prediction capacity but not adequate to supply the conventional processes, while the models for predicting ash content and HMF were not satisfactory. The developed models represent a low-cost tool to analyze the quality of honey, and contribute significantly to increasing the honey distribution and subsequently the economy of the region.

## 1. Introduction

Honey is a natural product, and a complex solution elaborated by honey bees. It is mainly composed of sugars (70–80%) and water (10–20%), and in minor quantities contains flavonoids, phenolic acids, vitamins, proteins, organic acids, lipids, carotenoids, minerals, and enzymes [1]. Honey has been used since ancient times as a food supplement for humans. Additionally, due to its content of phenolic compounds and flavones, it also has several beneficial health effects, which include prebiotic, antimicrobial, anticarcinogenic, antioxidant, antihypertensive, antibacterial, antifungal, anti-inflammatory, and analgesic effects [2,3,4]. The physical, chemical, and biological properties of honey depend on the type of flowers visited by the honey bees, and the soil where the nectar and pollen are collected. Other influences on its quality are the environmental and storage conditions, as well as the processing for its commercialization [5]. Therefore, quality control of honey represents an important concern for the beekeeping industry, since, on the one hand, it allows tracing of the geographical and botanical origin of the pollen (designation of origin), and, on the other hand, it allows identification of its possible adulteration during processing [6,7].

To classify and determine the honey’s quality, standards and methods have been established in the Codex Alimentarious [8], International Honey Commission (IHC) [9], and the Association of Official Analytical Chemists (AOAC) [10]. These standards specify the physical and chemical properties that must be evaluated to determine the honey’s quality. The traditional method to perform quality tests on honey involves the analysis of pollen grains contained in its sediments by light microscopy (melissopalynology) [6]. Other methods reported in the literature include chromatography techniques, stable carbon isotope radio analysis, and nuclear magnetic resonance [7,11]. The main drawbacks of all of them are their high cost, time consuming nature, requirement for specialists, and furthermore the fact that many of them are destructive. This has led to the development of analytical methods for the authentication of honey. In this sense, spectroscopy technology combined with chemometric tools represents a good alternative for the fast, reliable, and environmentally friendly quality control of honey samples. The above is due to the development of calibration models that can determine the concentration of a specific chemical species in a mixture of several components [12]. Among the most common chemometric techniques used in honey analysis are Principal Component Analysis (PCA), Hierarchical Clustering Analysis (HCA), Linear Discriminant Analysis (LDA), Partial Least Square (PLS), and Principal Component Regression (PCR) [13].

From the spectrometric techniques available, Raman spectrometry has suitable characteristics for food analysis, such as non-interference from water present in the sample with the Raman measurement, ease of sampling and measurement, and minimal fluorescence interference of the sample matrix variation. In recent years, analytic methods based on Raman spectrometry have been explored as an economic and rapid option to determine honey’s destination of origin [14,15,16,17]. Corvucci et al. [14] contrasted the ability to identify honey’s botanic origin using the melissopalynology technique compared to Raman spectroscopy coupled with multivariable analysis (PCA). The study considered honey samples from Italy, Eastern Europe, and Spain. According to the results, the discrimination of honey origin given by the two first principal components was improved from 85% to 99% using the analytical method. Frausto-Reyes et al. [15] determined the floral origin of honey produced by *Apis Mellifera*, applying Raman spectroscopy together with PCA. The study used 66 samples of both monofloral and polifloral honey collected from several regions of Mexico with different climate types. The use of the chemometric approach was adequate to classify the origin of the sample and the purity of the pollen with 90% accuracy. Jandrić et al. [16] presented a method for the authentication of floral origin honey produced in New Zealand. They combined Raman spectrometry, near infrared spectrometry, and Fourier-transform infrared spectroscopy for the analytical study of honey samples in the range between 200 to 12,000 cm^−1^. This approach was completed with the use of PLS for the development of chemometric models. The results showed a model fit (R^2^), a standard error of calibration (SEC), and standard error of prediction (SEP) of 85.0%, 0.219 and 0.315, respectively. Oroian and Ropciuc [17] applied Raman spectra analysis for the botanical authentication of 76 samples of honey from Romania. The use of this analytic method combined with LDA proved to be an excellent authentication tool, achieving 83.33% cross validation accuracy.

Similarly, the literature reports the use of Raman spectra analysis coupled with multi-variable modeling for the detection of external agents that affect the quality of honey [18,19,20,21,22]. Raman spectroscopy and chemometric models have been used to predict the concentration of glucose, fructose, sucrose, and maltose present in honey samples from Turkey and Greece [18]. The correlation between quantified sugar levels and Raman spectra was performed using both PLS and artificial neural networks (ANN). The statistical R^2^ for glucose, fructose, sucrose, and maltose were high, with 0.929, 0.930, 0.937, and 0.893 for PLS and 0.930, 0.931, 0.956, and 0.913 for ANN, indicating that both chemometric tools are efficient for the rapid analysis of sugar content. Oroian et al. [19] used Raman spectroscopy to detect honey adulterated with sugars (glucose, fructose, inverter sugar, hydrolyzed inulin syrup, and malt must). The study considered 900 samples with adulteration levels of 5, 10, 20, 30, 40, and 50%. Authentication of honey purity concentration was performed using PLS and PCR. The chemometric models developed showed good fit for both the calibration (R^2^_cal_ = 0.983) and validation (R^2^_val_ = 0.981) dataset, with low statistical errors (SEC = 0.009 and SEP = 0.103). Anjos et al. [20] evaluated the potential of Raman spectroscopy in the prediction of the physicochemical composition of *Lavandula* spp. monofloral honey. PLS models were used for the quantitative estimation, and the results were correlated with the values obtained using reference methods. Chemometric models were used for pH, sugar reduction, electrical conductivity, apparent sucrose, total phenol content, total flavonoid content, proline, and total acids, achieving R^2^_cal_ in the range of 0.973–0.99, R^2^_val_ in the range of 0.833–0.99, SEC in the range of 2.03–0.01, and SEP in the range of 1.71–0.01. In the study by Tahir et al. [21], Raman spectroscopy combined with PLS were applied to predict phenolic compounds and antioxidant activity in honey. It was found that the developed models based on Raman were superior to those established using NIR spectra, with R^2^_cal_ and R^2^_val_ > 90%, SEC < 1.2, and SEP < 1.7. Raman spectroscopy, and PLS-LDA modeling have also been used to determine the adulteration of Chinese honey with corn syrup [22]. The analysis considered adulteration samples in the range of 10, 20, and 40%. An accuracy prediction of 84.4% was obtained, indicating that combining PLS-LDA with Raman spectra is a potential technique for the detection of impure agents in honey.

In this paper, a study is presented to determine the physical-chemical properties of honey from the Mexican region of the Yucatan Peninsula. In this zone, beekeeping is an ancient activity, carried out since the pre-Columbian era by Mesoamerican cultures like the Maya, who already produced honey from apiaries with honey bees (*Melipona beecheii*) long before the arrival of the Spaniards [23]. After their conquest, the species *Apis mellifera* was introduced in Mexico, which proliferated and dispersed throughout the country due to its higher yields of honey. Currently, the Yucatan Peninsula (located in the south of the country and composed of the states of Yucatan, Campeche, and Quintana Roo) is one of the most fruitful regions for the development of beekeeping activity. This region is characterized by ecosystems with great flora diversity, producing nectars and pollen—many of them endemic—that produce honey with unique organoleptic, physical, and chemical properties; these characteristics make honey from this region very appreciated in national and international markets [24]. In this sense, Mayan beekeepers from the Yucatan Peninsula contribute approximately 35% of the national production. In the state of Campeche, there are 4030 honey producers that generate on average 5571 metric tons of honey per year; Campeche is the second honey producer region nationwide, only surpassed by Yucatan. Of the total produced in this region, 95% is exported, producing profits of up to 12 million US dollars and contributing to generating economic welfare for Mayan beekeepers [25,26,27]. Thus, the introduction of fast and low-cost tools to analyze the quality of the honey produced would contribute significantly to increasing distribution of this natural food, benefiting local beekeepers and the local economy.

Therefore, due to the economic importance of honey production in the state of Campeche, Mexico, the objective of this work was to develop chemometric models based on Raman spectroscopy for the quantification of the following physical and chemical properties: pH, moisture, total soluble solids (TSS), free acidity, lactonic acidity, total acidity, electrical conductivity (EC), Redox potential, hydroxymethylfurfural (HMF), and ash content. These chemometric models represent useful tools for the quality control of honey produced in the state of Campeche, by quickly and economically predicting the main physicochemical indicators.

## 2. Analysis of Results 

### 2.1. Raman Analysis

**Figure 1:**
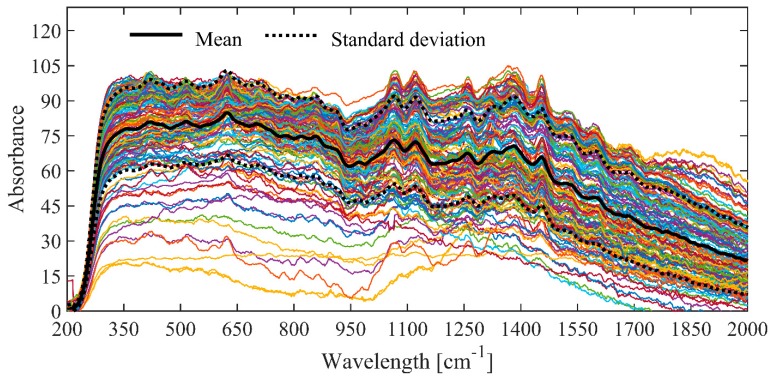
Raman spectral footprints of the honey collected in the various locations of Campeche.

Figure 1 shows that the Raman spectra obtained from the honey samples have spectral bands which cover the ranges of 330–404, 404–440, 440–510, 510–595, 595–691, 691–752, 770– 820, 820–1024, 1024–1094, 1094–1191, 1191–1262, 1262–1300, and 1300–1460 cm^−1^:

- Spectral region between 230–510 cm^−1^ are related to stretching and bending vibrations of the C-O, C-C-O and C-C-C that form the molecular structure of sugars [21].
- The region between 595–691 cm^−1^ is attributed to stretching vibrations of unsaturated rings present in HMF, carotenes, flavones, flavonoids, and polyphenols [22].
- The peak found between 691–752 cm^−1^ is assigned to stretching vibrations of C-O and C-C-O, and bending vibrations of O-C-O. On the other hand, the band between 770–917 cm^−1^ is a product of the stretching vibrations of the C-C and C-H present in glucose [28].
- Regarding the bands between 820–1024 cm^−1^, these correspond to deformation vibrations of C-H and methylene bonds –CH_2_–, as well as the bending vibrations of C-O-H [29].
- The peak present between 1024–1094 cm^−1^ is attributed to bending vibrations of the C-H and C-O-H bonds of sugars, and bending vibrations of the C-N bonds of amino acids and proteins [30].
- The band between 1094–1191 cm^−1^ is assigned to stretching vibrations of the C-O, C-O-C bonds of sugars, and the C-N bonds of proteins and amino acids [18].
- Finally, the spectral region between 1262–1300 cm^−1^ corresponds to vibrations of C-H and O-C-H, while the spectral bands of 1300–1460 cm^−1^ are due to bending and wobble vibrations of the functional groups CH and –OH [30].

### 2.2. Chemometric Models

#### 2.2.1. Chemometric Models to Predict pH, Free Acidity, Lactonic Acidity, and Total Acidity

The presence of organic acids, such as gluconic, phenolic, ascorbic, lactic, and metallic ions, causes honey to be slightly acidic by nature. The acidity may be increased due to chemical and biochemical changes that take place in the honey. For example, the glucose oxidase enzyme is capable of transforming glucose into gluconic acid; on the other hand, the ions of the alkaline earth elements can react to form phosphates, sulfates, and chlorides, as well as transform lactone into lactic acid [31]. To measure these chemical changes in honey, in the Codex Alimentarius [8], the pH, free acidity, lactonic acidity, and total acidity were established as quality control criteria. In this sense, free acidity is related to the concentration of organic acids in honey, where a maximum value of 50 meq kg^−1^ is established by the Codex Alimentarius.

Table 1 lists the values of the 10 physicochemical parameters determined for honey samples from the municipalities of the state of Campeche. As reported in the table, the pH of honey samples were in the range of 3.49 to 5.2, within the limit established by the Codex Alimentarius (minimum 3.40 and maximum 6.10). The minimum and maximum values of free acidity were detected between 22.5 and 35.1 meq kg^−1^, 4.15 y 9.45 meq kg^−1^ for lactonic acidity, and 28.67 a 38.28 meq kg^−1^ for total acidity. According to this, the values of the total acidity present in honey samples agree with the provisions of the Codex Alimentarius, indicating that the honey collected did not show significant degradation.

The variability in the pH, free acidity, lactonic acidity, and total acidity is represented in Table 1 by the standard deviation (σ). In this sense, the honey samples with the highest pH standard deviation were those from the municipalities of Calakmul and Holpechen, with ±0.23 and ±0.42, respectively. This variability is attributed to the diversity of melliferous flora present in the region (Figure 1), which belongs to the Calakmul biosphere reserve and houses more than 150 melliferous flowers, with important differences in their chemical composition [24,25]. On the other hand, honey samples that presented higher pH values (4.18–5.2) correspond to productions from the Tajonal and Mangle Negro plants, characterized by a higher concentration of sodium chloride. The Tajonal is a plant widely distributed in the state of Campeche, which is adapted to alkaline soils and is capable of growing near coastal areas, where a sea breeze is deposited on the flowers. Likewise, Mangle Negro grow in the coastal zone, on the banks of lagoons and estuaries that contain waters with high salinity; this contributes to the fresh honey from these flowers having low acidity due to the presence of sodium chloride.

Regarding total acidity, this presents standard deviations of ±5.44 meq kg^−1^ for honey samples from Calakmul and ±6.82 meq kg^−1^ in honey from Hopelchen. The free acidity for honey from the municipalities of Carmen has standard deviations ±4.41 meq kg^−1^ and ±4.47 meq kg^−1^ for those of Champotón, and ±5.13 meq kg^−1^ for Escarcega. The municipalities of Carmen, Champotón, and Escarcega are geographically are located in the west of the state of Campeche, a region characterized by lagoons, wetlands, rivers and estuaries that are conducive to the growth of melliferous plants such as Arbol de tinto, Pucté, Mangle, Cascarillo, and Ja’abin, among others. The honey of these floral species has a higher moisture content, which favors honey fermentation. On the other hand, the Hecelchacan honey samples showed a standard deviation of ±5.41 meq∙kg^−1^. This variability is attributed to the predominance in this region of melipona honey, which by its nature usually contains water concentrations above 20%, favoring the formation of organic acids by biochemical reactions.

**Figure 2:**
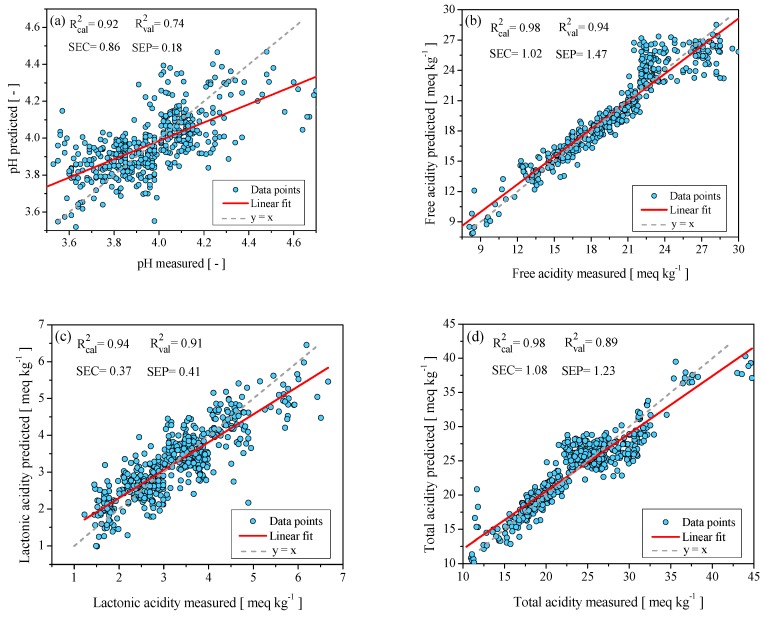
Chemometric models to predict: (**a**) pH; (**b**) free acidity; (**c**) lactonic acidity; (**d**) total acidity.

Based on the measurements obtained, chemometric models were created to predict pH, free acidity, lactonic acidity, and total acidity. Figure 2 shows the predictive behavior of the models, while Table 2 contains their statistical performances. The calibration model to predict the pH in honey of the state of Campeche exhibits a standard error of calibration SEC = 0.86 and standard error of prediction SEP = 0.18; likewise, it presents acceptable values for the coefficient correlation of calibration (R^2^_cal_ = 0.92) and the coefficient correlation of validation (R^2^_val_ = 0.74). These statistical values show that the chemometric model has an acceptable ability to predict the pH in honey. On the other hand, Student’s *t*-test with paired data at 95% confidence obtained t_c_ = 0.95, within the established confidence interval (t_v_ = ±1.65). Therefore, the chemometric model based on Near Infrared Spectroscopy (NIRS) has a good reliability but not enough to substitute the standardized method. The statistical values obtained in this work are similar to those reported by Cozzolino et al. [32], who obtained a chemometric model using Vis-NIRS spectroscopy to predict the pH of honey in Uruguay. They also reported values of SEC = 0.13, SEP = 0.21, R^2^_cal_ = 0.88, and R^2^_val_ = 0.70. On the other hand, Anjos et al. [20] reported statistical values of SEC = 0.12, SEP = 0.09, R^2^_val_ = 0.83, and R^2^_cal_ = 0.98 for a calibration model based on the FT-Raman spectroscopy used to predict the humidity percentage in Portuguese honey.

The chemometric model for predicting free acidity presented a standard error of calibration (SEC = 1.02), a standard error of prediction (SEP = 1.47), coefficient correlation of calibration (R^2^_cal_ = 0.98, and coefficient correlation of validation (R^2^_val_ = 0.94). These results indicate that the chemometric model successfully predicts the concentration of honey’s free acidity. The Student’s *t*-test of paired data (t_c_ = 0.64) for free acidity is within the confidence interval (t_v_ = ±1.65), indicating that there are no differences in the prediction capacity of the developed chemometric model with respect to the standard method established in the Codex Alimentarius [8]. In previous studies, such as the one carried out by Ruoff et al. [33], the following statistical values were reported for a chemometric model based on NIRS spectroscopy to predict free acidity in Swiss honey: a standard error of calibration (SEC = 2.01), standard error of prediction (SEP = 2.0), and coefficient correlation of validation (R^2^_val_ = 0.737).

With regards to the chemometric model for predicting lactonic acidity in Campechean honey, it showed good predictive capacity, since the values of cross-validation and external validation, along with the standard error of calibration and standard error of prediction, were small (SEC = 0.37; SEP = 0.41), with the following coefficient correlation of calibration and coefficient correlation of validation (R^2^_cal_ = 0.94; R^2^_val_ = 0.91). For the Student’s *t*-test of paired data (t_c_ = 0.69) at 95% confidence, the value obtained is in the confidence interval (t_v_ = ±1.65), so there are no differences in the prediction capacity of lactonic acidity between the obtained chemometric model and the standard method [8].

Finally, the chemometric model to predict total acidity in Campeche honey showed a high coefficient correlation in the cross-validation (R^2^_cal_ = 0.98) and coefficient correlation in the external validation (R^2^_val_ = 0.89), as well as low values of standard error of calibration (SEC = 1.18) and standard error of external validation (SEP = 1.23). Moreover, the Student’s *t*-test of paired data (t_c_ = 0.75) is in the confidence interval (t_v_ = ± 1.65), which demonstrates that the chemometric model is as reliable as the standardized method. Comparing the obtained results with those reported by Anjos et al. [20] for an FT-Raman spectroscopy calibration model to predict the acidity total in Portuguese honey, similar values were observed (SEC = 0.22; SEP = 0.28; R^2^_cal_ = 0.99; R^2^_val_ = 0.99).

In Figure 2, it can be seen that the experimental data of the pH, free acidity, lactonic acidity, and total acidity of the honey samples show a certain degree of dispersion compared to the chemometric model predictions. This can be attributed to the following factors: first, in the state of Campeche, several tropical forests are located that give rise to a great diversity of honey blooms; previous works have identified more than 150 blooms in the area of study [24,25,26]. Thus, the honeys produced in the region are multifloral, giving rise to a wide variety of physical and chemical properties. Second, the geographical origins where the honey samples were collected—specifically in the east of the state of Campeche, in the municipalities of Carmen, Palizada, Escarcega, and Champotón—are characterized by the presence of rivers, lagoons, wetlands, and swamps. These soils are rich in organic matter and have an acidic pH, which contribute to the development of a great diversity of melliferous flora, such as: Tahonal, Ja’abin, Pukte, huano, Xtabentum, Palo Tinto, hulub, Suuk chak lol, Box káatsim, Bohom, Susuk, cascarillo and mangle negro. Flowers from these botanical origins produce nectar with high concentrations of moisture, which is transferred to the honey [26]. The presence of a high percentage of moisture in honey favors biochemical and chemical reactions—for example, the formation of gluconic acid from glucose and the formation of inorganic acids due to the reaction of water with anions and cations present in honey. This means that honey samples collected in these locations show greater variability in pH, free acidity, lactonic acidity, and total acidity [34,35].

#### 2.2.2. Chemometric Model to Predict Electrical Conductivity, Redox Potential, Moisture, and TSS

**Figure 3:**
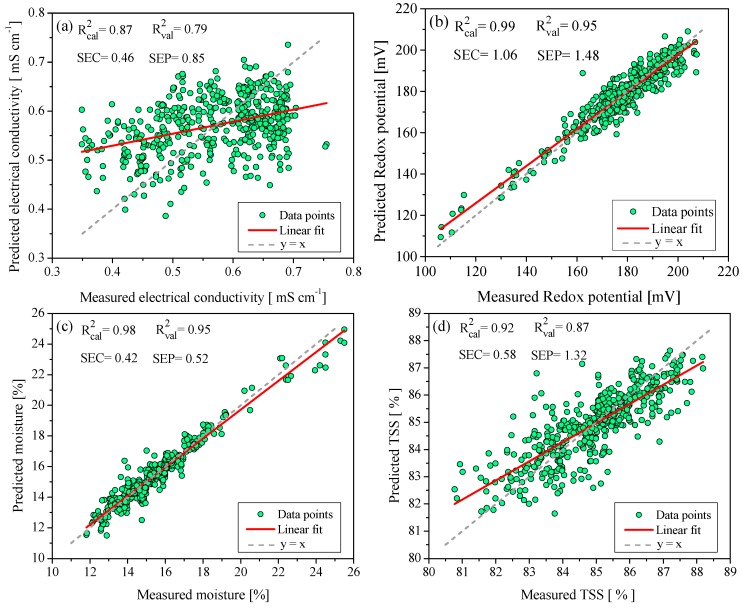
Chemometric models to predict: (**a**) electrical conductivity; (**b**) Redox potential; (**c**) moisture; (**d**) TSS.

Electrical conductivity is a parameter used to determine the geographical origin of honey. This is related to the content of ashes, organic acids, and dissolved mineral salts; the higher the concentration of these compounds in honey, the greater the value of the electrical conductivity [36]. In this sense, the diverse honey samples from the state of Campeche presented values between 0.28–0.75 mS cm^−1^, which is below the maximum allowed limit (0.80 mS cm^−1^ [8]). The chemometric model for this physicochemical property had a standard cross-validation error and an external validation error of 0.46 and 0.85, respectively. Moreover, the regression coefficients obtained were R^2^_cal_ = 0.87 and R^2^_val_ = 0.79. Nevertheless, the R^2^_val_ value indicates an acceptable model fit, but are not significant for our propose. In Figure 3a, a noticeable dispersion between the experimental data of the electrical conductivity with respect to the chemometric model is observed. This is attributed to the significant differences in organic matter, salinity content, and carbonates in the soils of the locations where the honey samples were collected. Another cause is the diversity of the honey flora, which contributed to the variation in the content of organic acids in the honeys [24,31].

Comparing the results obtained with previous works, these present slightly lower values than those reported by Anjos et al. [20], who built a chemometric model based on FT-Raman for Portuguese honey. They reported values of calibration errors and external validation of (SEC = 0.01; SEP = 0.01), and coefficients of determination (R^2^_c__al_ = 0.92.8; R^2^_r__val_ = 0.938). Nonetheless, the results obtained in our study are similar to those reported by Ruoff et al. [33] for a chemometric model based on NIRS spectroscopy to predict electrical conductivity in Swiss honeys (R^2^_c__al_ = 0.794 and R^2^_r__val_ = 0.87); and with the data reported by Cozzolino et al. [32] for a calibration model of Uruguayan honey (R^2^_c__al_ = 0.83 and R^2^_r__val_ = 0.80).

On the other hand, honey contains chemical substances dissolved in low concentrations of organic acids, mineral salts, and polyphenols; polyphenols are molecules that contain unsaturated bonds in their chemical structure, and develop a very important function since they are antioxidants; these substances have the property of trapping free radicals generated in biochemical reactions. When honey undergoes cooking processes or remains stored for a long period, the aforementioned substances may undergo oxide–reduction reactions, causing changes in their molecular structure and modifications in the properties of honey. These chemical changes can be monitored using the Redox potential to determine the degree of oxidation. Because the Redox potential can be used as a quality control parameter, it was analyzed in Campeche honeys. The results indicate Redox potential values with a minimum of 133.1 mV and a maximum of 207.2 mV; the difference in these results is attributed to the composition of each bloom. The chemometric model had calibration and validation errors (SEC = 1.06; SEP = 1.48), and high values in the calibration and external validation coefficients (R^2^_c__al_ = 0.99; R^2^_r__val_ = 0.95). The reliability of the model was also confirmed by a Student’s *t*-test of paired data, with a value of t_c_ = 0.545 between t_v_ = ±1.65 at 95% confidence, so the model has a good predictive capacity.

With regards to moisture, a maximum content of 20% was defined in the Codex Alimentarious [8]. This is because an excess of moisture favors the fermentation of sugars, causing the formation of undesirable organic acids that affect the organoleptic properties [37]. The moisture content in honey depends on several factors, such as floral origin, harvest time, climate change, maturity degree of the honey, and improper handling of the honey by beekeepers [38]. The analyzed honey samples presented humidity values between 11.81–25.81%; some samples showed humidity concentrations above 20% because the honey came from tree blooms located in wetlands, near rivers, and near estuaries. In addition, some samples were from melipona honey, that, by nature, contains high concentrations of moisture [39]. The chemometric model to predict moisture in honey presented SEC = 0.42, SEP = 0.52, R^2^_c__al_ = 0.98, and R^2^_val_ = 0.97. Additionally, t_c_ = 0.41 was obtained in the Student’s *t*-test, which indicates that the chemometric model correctly predicts moisture in the honey. The presented results are similar to those reported by Lichtenberg et al. [40] for a predictive model of moisture in German honeys (R^2^_c__al_ = 0.73 and σ = ±1.22). Likewise, it is consistent with what was reported by García et al. (2000) regarding a chemometric model to predict moisture in honey from the region of Galicia, Spain (SEC = 0.12, SEP = 0.15, and R^2^_c__al_ = 0.98); and with Cozzolino et al. [32], who reported values of (SEC = 2.7, SEP = 3.1, R^2^_c__al_ = 0.96, and R^2^_val_ = 0.94) for a calibration model focused on predicting moisture content in Uruguayan honey.

The principal component of honey is sugar; honey contains a mixture of sugars, mainly fructose, glucose, sucrose, maltose, and melezitose. Glucose and fructose are the ones that are found in the highest proportion and can represent up to 95% of the sugar content [41]. The honey samples collected in the state of Campeche exhibited total sugar concentrations between 74.19–88.19% w; some samples presented concentrations below 80 ° Brix [8] due to a higher moisture concentration. The chemometric model developed to predict TSS showed the following statistical results: SEC = 0.58; SEP = 1.32; R^2^_cal_ = 0.92; R^2^_val_ = 0.87. A Student’s *t*-test with a value of t_c_ = 0.28 was in the range t_v_ = ±1.65 at 95% reliability, which shows that the model for predicting TSS has an acceptable prediction capacity but not adequate to supply the referenced method. The results obtained in this work were similar to those reported by Mignani et al. [42], who built chemometric models based on Raman spectroscopy to predict glucose and fructose concentrations in Italian honeys (SEC = 7.3; SEP = 11; R^2^_cal_ = 0.96, R^2^_val_ = 0.92) and (SEC = 5.5; SEP = 7.6; R^2^_cal_ = 0.89, R^2^_val_ = 0.82). Likewise, Özbalci et al. [18] developed calibration models based on Raman spectroscopy to predict glucose and fructose concentrations in Turkish honeys, reporting the following values (SEC = 0.51; SEP = 2.75; R^2^_cal_ = 0.98; R^2^_val_ = 0.96). Complementing this, Anjos et al. [20] reported statistical results (SEC = 0.34, SEP = 0.39, R^2^_cal_ = 0.99, R^2^_val_ = 0.99) for a predictive calibration model of reducing sugars in Portuguese honey. The comparisons between values predicted by the chemometric models presented in this section and their respective experimental values are shown in Figure 3.

#### 2.2.3. Chemometric Model to Predict Content of HMF and Ashes

As presented in Table 2, the chemometric models to predict ash percentage and HMF content presented low coefficients of determination in cross-validation and external validation (R^2^_c__al_ = 0.78, R^2^_val_ = 0.21; and R^2^_c__al_ = 0.82, R^2^_val_ = 0.56). The above indicates that the models are not suitable for the prediction of these chemometric properties.

### 2.3. Analysis of the PLS loadings

**Figure 4:**
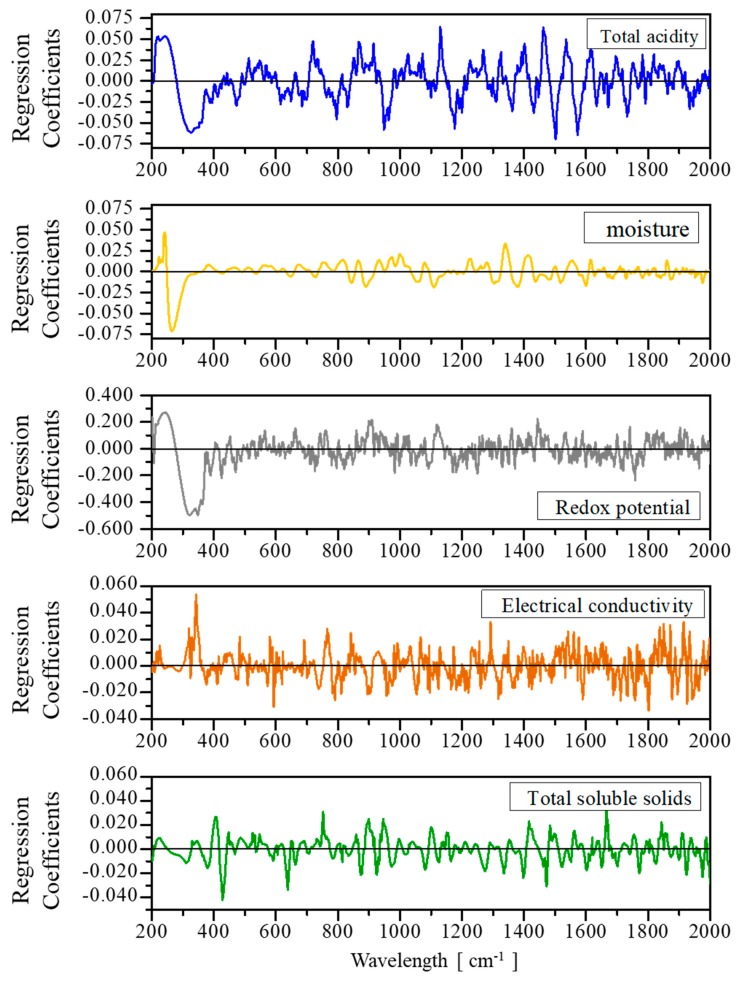
Regression models in the Raman region obtained to predict physical and chemical properties of honey from the state of Campeche.

The PLS loading for total acidity, electrical conductivity, Redox potential, humidity and TSS (Figure 4) present six spectral regions (200–600 cm^−1^, 630–790 cm^−1^, 870–1000 cm^−1^, 1080 –1200 cm^−1^, 1400–1570 cm^−1^, and 1750–1880 cm^−1^) that provide useful chemical information for the development of their respective predictive chemometric models. The first spectral band (between 200–400 cm^−1^) has a positive and negative contribution in the PLS loading. The chemical information provided by this region is related to stretching, bending, and deformation vibrations of C-O, C-C-O, C-C-C and C=O, which form the skeleton of sugar molecules, organic acids, phenolic compounds, and flavonoids. Here, breaks of functional groups and of the sugar backbone can occur due to oxide–reduction reactions; for example, in the transformation of glucose into gluconic acid, fermentation reactions for the production of alcohols and carboxylic acids and the cyclization of fructose produce HMF. These chemical changes in the honey collected would reflect variations in total acidity, pH, Redox potential, and electrical conductivity with respect to time. The band at 630–790 cm^−1^ provides chemical information of the cyclic and alicyclic rings that make up the molecules of HMF, carotenes, flavonols, flavanones, and flavones, among other phenolic compounds. The chemical information related to the band between 870–1000 cm^−1^ is attributed to stretching, bending and deformation vibrations of the C-C, C-H, C-H, –CH_2_–, and C-O-H bonds present in the sugars. The Raman region between 1080–1200 cm^−1^ provides information on protein and carbohydrate content in honey, due to stretching vibrations of C-O, C-O-C, C-N carbohydrate, and protein bonds. The region between 1400–1570 cm^−1^ provides chemical information due to bending and wobble vibrations of CH, O-C-H and –OH functional groups present in sugar molecules, and –OH in the water molecules. Finally, the concentrations of moisture, fructose, glucose and moisture in honey are related to stretching vibrations of the unsaturated bonds C=O in fructose and CH=O in glucose, and deformation vibrations –OH of water, which are present in the Raman spectrum between 1750–1880 cm^−1^.

## 3. Materials and Methods

### 3.1. Honey Samples

**Figure 5:**
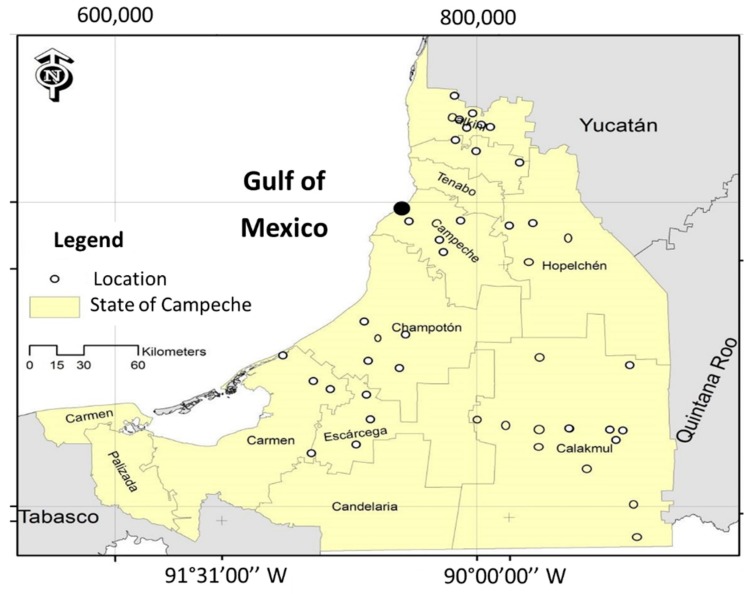
Honey producing communities in the state of Campeche.

A total of 189 honey samples were supplied directly from Mayan beekeepers of the state of Campeche, Mexico. The samples were collected between February and June of 2014 and 2015. From the total samples, 175 corresponded to *Apis mellifera* and 14 to *Melipona beecheii*. Figure 5 illustrates the geographical region where the honey samples were collected, which includes the locations of Calakmul (40 samples), Calkiní (14 samples), Campeche (26 samples), Champotón (34 samples), Escárcega (20 samples), Hecelchakán (4 samples), Hopelchén (22 samples), and Sabancuy (29 samples). The predominant floral origin of the honeys was determined according to information provided by the Mayan beekeepers, and included the following: Tahonal (*Viguiera dentata*), Tsíitsilche (*Gymnopodium floribundum*), Ja’abin (*Piscidia piscipula*), Tzalam (*Lysiloma latisiliquum*), Pukte (*Bucida buceras*), Xa’an, huano (*Sabal yapa*), Xtabentum (*Turbina corymbosa*), Palo Tinto (*Haematoxylum campechianum*), Chéechem (*Metopium brownei*), Hulub (*Bravaisia berlandieriana*), Chakàah (*Bursera simaruba*), Suuk, chak lol (*Salvia coccínea*), Box káatsim (*Acacia gaumeri*), Bohom (*Cordia gerascanthus*), Kitim che’ (*Caesalpinia gaumeri*), Susuk (*Dyphisa carthagenensis*), Cascarillo (*Erythroxylum confusum*), Machiche (*Lonchocarpus castilloi*) and Mangle negro (*Avicennia germinans*).

### 3.2. Physicochemical Analysis

The physical-chemical properties of the honey samples were determined according to standards and methods established by Codex Alimentarious [8], International Honey Commission [9], and the Association of Official Analytical Chemists [10]. The honey properties studied were pH, moisture, TSS, free acidity, lactonic acidity, total acidity, EC, Redox potential, HMF, and ash content. The chemical reagents used were standard hydrochloric acid (HCl) solution at 0.05 N, standard sodium hydroxide (NaOH) solution at 0.05 N, deionized water, acetone, buffer solutions, sodium bisulfite (Fermont, Canada), and the reagents Carrez I and Carrez II (Sigma-Aldrich, Saint Louis, MO, USA); all of them of analytical grade. A detailed description of the procedure for obtaining each physical-chemical property is given below.

#### 3.2.1. Moisture and Total Soluble Solid

Moisture and TSS were measured by the refractometric method. One gram of honey was analyzed in an Atago refractometer model PAL-22S (Atago, Tokio, Japan) at 25 °C; TSS was expressed in Brix°, whereas moisture percentage (g/100 g honey) was given according to the method established in [43].

#### 3.2.2. pH, Free Acidity, Lactonic Acidity, and Total Acidity

To determine the pH, 10.0 g of honey was dissolved in 75 mL of deionized water (free CO_2_). The solution was analyzed by using a Thermo Scientific brand pH meter (Orion Star A211, Waltham, MA, USA), previously calibrated with standard buffer solutions at pH values of 4–7 and 7–10, respectively. The honey solution was titrated with 0.05 N NaOH until it reached a pH of 8.5 to obtain the free acidity value. Lactonic acidity was determined by adding 10 mL of 0.05 N NaOH to the sample, and then titrating with 0.05 N HCl to return the pH to 8.3. Finally, the total acidity was obtained as the sum of the free acidity and lactonic acidity values, expressed in meq∙kg^−1^ [44].

#### 3.2.3. Electrical Conductivity and Redox Potential

The electrical conductivity and Redox potential were measured using a conductivity meter (Thermo Scientific, Waltham, MA, USA), which analyzed a solution composed of 20 g of honey dissolved in 100 mL of deionized water (free CO_2_). Measurements were made at 20 °C and the results were expressed in mS∙cm^−1^ for electrical conductivity and mV for Redox potential [45,46].

#### 3.2.4. Ash Content and Hydroxymethylfurfural

The determination of ash content was conducted by incineration [47]. Two grams of honey was placed in a crucible and heated in a Lindberg/Blue muffle furnace (Thermo Fisher Scientific, USA) at 650 °C for 6 h. Carbon content results were expressed in g/100 g honey. On the other hand, HMF content was measured based on the standard method [10]. Five grams of honey was dissolved with 25 mL of deionized water (free CO_2_) in an (250 mL) Erlenmeyer flask. The solution was clarified by adding 0.5 mL of Carrez I and Carrez II reagents, up to 50 mL. The solution was filtered using Watman paper (No. 42), and subsequently treated with a sodium bisulfite solution. The absorbance was determined on a UV-visible spectrophotometer (DR6000, HACH, Loveland, CO, USA) at wavelengths of 284 and 338 nm. HMF concentration was expressed in mg∙kg^−1^.

### 3.3. Raman Analysis

Honey samples were analyzed in triplicate using a Raman QE65000 spectrometer (Ocean Optics, Edinburgh, UK) equipped with a symmetric crossed Czerny-Turner optical bench, 101 mm focal length, an RPB 785 fiber optic prove, and Hamamatsu S7031-1006 detector with a spectral range between 780–940 nm. The spectrometer was operated with the SPECTRA SUIT software (version 2.0.162, Ocean Optics, Edinburgh, UK) to establish the interface between the computer and the Raman equipment. To perform the analysis of the samples, 30 mL of honey was deposited in an amber glass bottle and subsequently a laser beam was applied at 785 nm with a power of 20 mW for 10 s. All Raman spectra were collected in the range of 0 to 2200 cm^−1^ at 25 °C with a spectral resolution of 1.55 cm^−1^. The data between 0–200 cm^−1^ and 2001–2200 cm^−1^ were omitted because they had higher spectral noise. Therefore, the spectral data between 201–2000 cm^−1^ was used.

### 3.4. Chemometric Model Development

**Figure 6:**
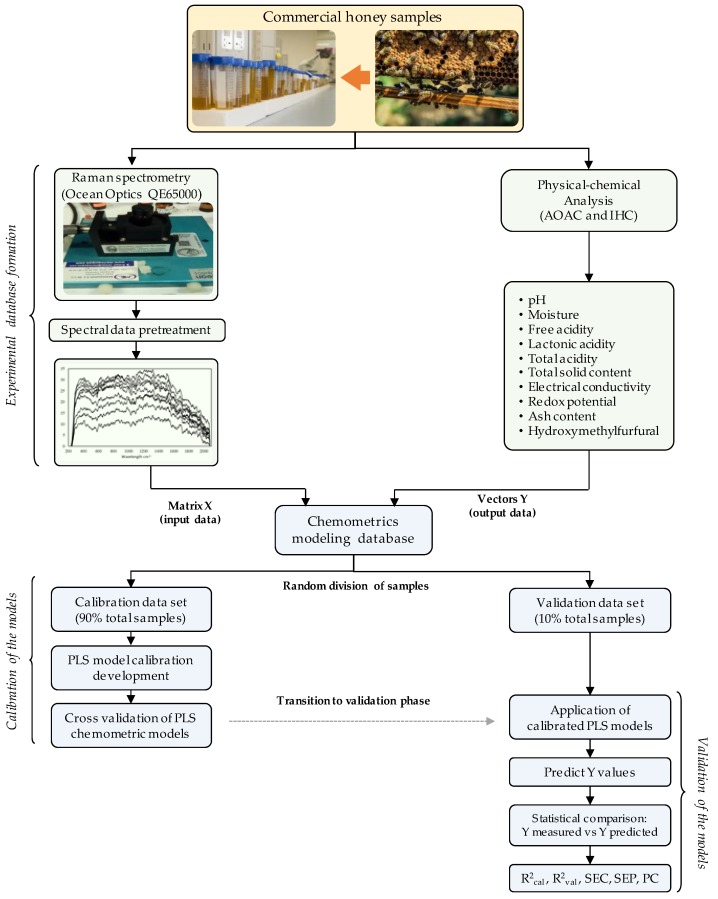
Schematic diagram of the development and evaluation process of the 10 chemometric models for the estimation of physicochemical properties of honey produced in the region of Campeche, Mexico.

For the development of the chemometric models, an experimental database was created employing the Raman absorbance (matrix X) and the physical-chemical properties of the analyzed honey samples (vectors Y). Raman analysis results were converted into a data matrix using Microsoft Excel 2013 (Microsoft, Redmond, WA, USA) composed of 900 wavelength values and 567 honey samples (510,300 absorbance samples). The data matrix was transposed and exported to the Pirouette V. 4.5 Software (Infometrix, Bogota, Colombia) to be correlated with each of the physicochemical properties. For the construction of chemometric models, partial least square (PLS) regression was used. In order to minimize spectral noise and errors in the development of the chemometric models, the following mathematical and statistical treatments were applied: auto-scaling or centering and subsequently the treatments baseline correction, smoothing, data normalization, first-order derivation, alignment, Log10 analysis, and Standard Normal Variate (SNV) were performed. To determine the predictability of the models developed, a cross-validation was performed (five out) using 90% of the data. Subsequently, an external validation was carried out with the remaining 10% of the data, which were not used in the construction of the chemometric models. The division of the database for the external calibration and validation processes was carried out by the software Pirouette, implementing the Kennard–Stone selection algorithm [33]. The statistical indicators used during the validation phase were: standard error of calibration (SEC), standard error of prediction (SEP), coefficient correlation of calibration (R^2^_cal_) and coefficient correlation of validation (R^2^_val_), and Student’s *t*-test of paired data [33,34]. Figure 6 illustrates the computational procedure for the development of the chemometric models.

## 4. Conclusions

In this work, it has been demonstrated that the Raman technique is an analytical tool that has advantages over other conventional techniques for the analysis of honey, since it is friendly to the environment and does not use chemical reagents, obtaining results in less time. Furthermore, it has been demonstrated that chemometric modeling based on Raman technology allows the development of numerical models and good capacity of predicting humidity, free acidity, lactonic acidity, total acidity, and Redox potential for Campechean honeys. The statistical parameters used to evaluate the predictability of each chemometric model show an accuracy similar to the conventional methods established in the standards, with the advantage that they are faster and do not use chemical reagents, so they are more environmentally friendly. Chemometric models to predict the content of HMF and ashes did not achieve good predictive capacity, which can be attributed to the fact that these chemical components are at very low concentrations in honey.

According to the study, the chemometric models that presented adequate prediction results represent an interesting alternative to be used in the development of intelligent portable laboratories that facilitate beekeepers in the region to analyze said chemometric properties at the site. Thus, the models presented represent a low-cost option to contribute significantly to the economic development of the honey industry in the region.

## Figures and Tables

**Table 1:** Results obtained for the different physical and chemical parameters of honey from the municipalities of the state of Campeche.

Property	Mean ± σ	Minimum	Maximum	Mean ± σ	Minimum	Maximum
	*Calakmul*			*Calkini*		
pH	4.01 ± 0.23	3.66	5.11	4.08 ± 0.17	3.80	4.77
Free acidity	21.16 ± 5.03	8.12	32.53	19.79 ± 3.03	15.52	25.51
Lactonic acidity	2.96 ± 1.001	1.23	5.78	2.77 ± 0.84	1.47	4.27
Total acidity	24.17 ± 5.44	11.55	36.78	22.51 ± 3.31	18.25	28.67
Electric conductivity	0.58 ± 0.08	0.35	0.69	0.61 ± 0.05	0.49	0.68
Redox potential	181.94 ± 13.91	133.1	207.2	173.54 ± 8.87	161.6	198.1
Moisture	14.98 ± 1.42	11.81	17.66	12.21 ± 2.27	12.29	16.66
TSS	85.02 ± 1.41	82.37	88.19	85.79 ± 1.09	83.34	87.71
Ash content	0.14 ± 0.06	0.018	0.42	0.143 ± 0.14	0.09	0.21
HMF	2.87 ± 1.33	1.27	5.89	2.31 ± 0.75	1.46	4.35
	*Campeche*			*Carmen*		
pH	3.95 ± 0.16	3.49	4.18	3.97 ± 0.14	3.64	4.25
Free acidity	17.03 ± 3.52	12.39	26.1	21.22 ± 4.19	8.01	28.53
Lactonic acidity	2.51 ± 0.68	1.47	4.15	3.09 ± 1.08	1.23	5.78
Total acidity	19.53 ± 3.81	14.17	29.65	24.32 ± 4.41	11.45	31.34
Electric conductivity	0.48 ± 0.08	0.28	0.69	0.57 ± 0.08	0.35	0.69
Redox potential	177.49 ± 9.89	151.3	204.2	186.23 ± 8.41	170.1	207.4
Moisture	15.25 ± 3.11	12.76	24.6	15.02 ± 1.53	11.81	17.66
TSS	84.74 ± 3.11	75.42	87.24	84.98 ± 1.53	82.34	88.19
Ash content	0.13 ± 0.018	0.08	0.16	0.14 ± 0.09	0.02	0.88
HMF	2.12 ± 0.46	1.52	3.53	2.98 ± 1.43	1.27	5.89
	*Champotón*			*Escarcega*		
pH	3.78 ± 0.18	3.55	4.23	3.85 ± 0.17	3.62	4.31
Free acidity	22.81 ± 4.26	11.9	32.5	22.72 ± 5.11	13.5	31.5
Lactonic acidity	3.59 ± 0.78	2.37	5.98	3.51 ± 0.62	1.78	4.37
Total acidity	26.41 ± 4.47	17.01	38.28	26.23 ± 5.13	17.07	35.59
Electric conductivity	0.54 ± 0.11	0.36	0.69	0.58 ± 0.12	0.35	0.755
Redox potential	189.03 ± 11.39	165.4	202.6	172.52 ± 9.38	146.1	185.8
Moisture	16.9 ± 3.11	13.32	25.81	15.16 ± 0.88	13.65	16.89
TSS	83.01 ± 3.11	74.2	86.36	84.83 ± 0.88	83.11	86.35
Ash content	0.14 ± 0.03	0.11	0.17	0.13 ± 0.02	0.068	0.18
HMF	3.34 ± 1.32	1.57	6.39	2.34 ± 1.44	1.57	4.89
	*Hecelchacan*			*Hopelchén*		
pH	4.09 ± 0.09	3.91	4.21	4.34 ± 0.42	3.51	5.2
Free acidity	17.78 ± 3.06	16.85	22.5	16.64 ± 6.95	6.5	35.1
Lactonic acidity	5.14 ± 2.48	3.19	9.45	3.44 ± 0.91	1.67	5.92
Total acidity	22.93 ± 5.41	21.07	31.95	20.08 ± 6.82	10.41	37.77
Electric conductivity	0.61 ± 0.056	0.51	0.659	0.59 ± 0.08	0.44	0.71
Redox potential	177.49 ± 14.34	167.5	202.1	153.93 ± 22.21	105.6	198.2
Moisture	17.09 ± 3.19	15.17	22.67	14.72 ± 1.23	12.43	17.4
TSS	82.85 ± 3.16	77.33	85.45	85.27 ± 1.23	82.6	87.57
Ash content	0.13 ± 0.015	0.11	0.14	0.14 ± 0.03	0.05	0.21
HMF	2.89 ± 0.265	2.39	3.27	3.18 ± 0.95	1.56	5.78

**Table 2:** Values of the statistical parameters obtained in cross-validation and external validation to determine the capacity predictability of each chemometric model.

Properties	Units	Calibration LVs	SEC	R^2^_cal_	Validation LVs	SEP	R^2^_val_
pH	-	5	0.86	0.92	4	0.18	0.743
Free acidity	meq kg^−1^	6	1.02	0.98	6	1.47	0.935
Lactonic acidity	meq kg^−1^	6	0.37	0.94	7	0.41	0.911
Total acidity	Meq kg^−1^	6	1.08	0.98	4	1.23	0.897
Electrical conductivity	mS cm^−1^	6	0.46	0.87	4	0.85	0.79
Redox potential	*mV*	7	1.06	0.99	8	1.48	0.95
Moisture	%	6	0.42	0.98	9	0.52	0.95
TSS	%	6	0.58	0.92	6	1.32	0.87
Ash content	%	6	1.21	0.78	6	2.54	0.21
HMF	mg kg^−1^	7	0.76	0.82	8	1.73	0.63

## Abbreviations

**σ**	Standard deviation
R^2^_cal_	Coefficient of determination of cross-validation
R^2^_val_	Coefficient of determination for external validation
t_c_	Student’s *t*-test confidence value
t_v_	Student’s *t*-test external validation value
X	Raman spectroscopy matrix data
Y	Vector value of a physicochemical property
ANN	Artificial Neural Networks
AOAC	Association of Official Analytical Chemists
EC	Electrical conductivity
FT	Fourier transform
HCA	Hierarchical Clustering Analysis
HMF	hydroxymethylfurfural
IHC	International Honey Commission
LDA	Linear Discriminant Analysis
LVs	Latent variables
NIRS	Near infrared spectroscopy
PCA	Principal Component Analysis
PCR	Principal Component Regression
PLS	Partial Least Square
SEC	Standard error of calibration
SEP	Standard error of prediction
SNV	Standard Normal Variate
TSS	Total soluble solids
